# *In Silico* Analyses of Primers Used to Detect the Pathogenicity Genes of *Vibrio cholerae*

**DOI:** 10.1264/jsme2.ME11317

**Published:** 2012-05-17

**Authors:** Julien Gardès, Olivier Croce, Richard Christen

**Affiliations:** 1 Université de Nice Sophia-Antipolis, Parc Valrose, Centre de Biochimie, F 06108 Nice, France; 2 CNRS UMR 6543, Institut de Biologie du Développement et Cancer, Parc Valrose, Centre de Biochimie, F 06108 Nice, France

**Keywords:** primers, *Vibrio cholerae*, virulence genes

## Abstract

In *Vibrio cholerae*, the etiological agent of cholera, most of the virulence genes are located in two pathogenicity islands, named TCP (Toxin-Co-regulated Pilus) and CTX (Cholera ToXins). For each *V. cholerae* pathogenicity gene, we retrieved every primer published since 1990 and every known allele in order to perform a complete *in silico* survey and assess the quality of the PCR primers used for amplification of these genes. Primers with a melting temperature in the range 55–60°C against any target sequence were considered valid. Our survey clearly revealed that two thirds of the published primers are not able to properly detect every genetic variant of the target genes. Moreover, the quality of primers did not improve with time. Their lifetime, *i.e.* the number of times they were cited in the literature, is also not a factor allowing the selection of valid primers. We were able to improve some primers or design new primers for the few cases where no valid primer was found. In conclusion, many published primers should be avoided or improved for use in molecular detection tests, in order to improve and perfect specificity and coverage. This study suggests that bioinformatic analyses are important to validate the choice of primers.

Since the first known cholera epidemics in India’s Ganges delta in 1817, this pathogen has swept across the globe in several worldwide pandemics, afflicting hundreds of millions of people and killing more than 70 percent of its victims within hours if left untreated. This pandemic continues, with the latest large outbreak in earthquake-ravaged Haiti, where a cholera epidemic occurred after a reported absence of some 100 years (13). Historically and for most people, cholera is seen as a disease of filth carried in sewage. However, research on cholera’s natural habitat and links to the climate have now led to the understanding of this disease as one driven just as much by environment, hydrology, and weather patterns as by poor sanitation. As temperatures continue to rise, cholera outbreaks may become increasingly common, with the bacteria growing more rapidly in warmer waters (35, 46).

Analyses of pathogenicity genes are an important tool for the diagnosis and treatment of infectious diseases. Amplifications using the polymerase chain reaction (PCR) and specific primers are often used to detect and analyze these genes; however, the sensitivity and specificity of a PCR reaction depend upon using good primers. Primers need to have a melting temperature (Tm) above 55°C (1) in order to be specific, to bind to every possible allele of a given gene and not to bind to non-target genes. In addition, secondary structures should be avoided (GC-clamp, hairpins, intramolecular interactions and finally self- or hetero-dimerization).

*Vibrio cholerae* is the etiological agent of cholera, a severe bacterial infection of the small intestine, and a major cause of death in developing countries. This bacterium lives in aquatic ecosystems and is often associated with copepods (14, 44, 45). The pathogenicity genes of *V. cholerae* are interesting targets to detect and study *V. cholerae* infections. Most of these genes are located in two pathogenicity islands, named TCP (Toxin-Co-regulated Pilus) and CTX (Cholera ToXins), organized as prophages (49, 75). TCP contains a cluster of genes involved in host adhesion via *pili*, while CTX genes are involved in the synthesis of the cholera toxin (25). Although the mechanisms of transfer are not still very well understood, these pathogenicity islands are known to be exchanged among strains of *V. cholerae*(52) and even with closely related species such as *V. mimicus*(77). Several *in silico* or “wet-biology” studies of the efficiency of PCR primers have been published, but they mostly analyzed the universal ribosomal RNA genes (3, 16, 27, 39–41, 43, 47, 53, 76) or housekeeping genes (56, 61, 65, 69), and no study is available for *V. cholerae*(8).

For each of the genes located in these two pathogenicity islands, we retrieved every published primer and every known allele in order to perform a complete *in silico* survey and assess the quality of the PCR primers used since 1990, the date of the earliest publication retrieved (51). Primers with a Tm above 55°C against any target sequence were considered valid for detection. Our results demonstrate that invalid primers have been published about twice more frequently than good primers, even in recent years. Also, the lifetime of a primer (as assessed by citations over years) is not related to its quality, since several invalid primers have been used for more than 15 years.

## Materials and Methods

### Ethics Statement: this study did not involve living beings or any biological samples

Every protein coding the DNA sequence belonging to the *Vibrio* genus was collected using the ACNUC database and its retrieval system (36). The ACNUC database has the advantage of (i) automatically extracting subsequences from large genomic sequences, and (ii) allowing precise searches using a combination of keywords separated by spaces, the use of a text file containing a list of keywords, of sequences according to cellular location, and the type of sequences (CDS, mRNA, rRNA, *etc.*). Then, tBLASTx analyses (with some optimized options such as the length of the word (w) as 3, the deactivation of filters, and the visualization of 1500 sequences maximum) were performed with a reference sequence, selected from a complete genome sequence, for each pathogenicity gene in order to retrieve similar sequences. The pathogenicity genes correspond to the 32 well-characterized genes of the two pathogenicity islands of *V. cholerae*(49, 75). A keyword search was also used to complement the similarity search. Using a word or a list of words describing a pathogenicity gene, the list of keywords used to annotate the gene features (proteins) was retrieved by our program reading the gene entries under the EMBL format. A recursive program was used to identify every alternate gene and protein name. These steps were repeated until no new keyword was found for the annotation of a given pathogenicity gene or gene product. Unfortunately, several problems due to misspellings or errors in annotations prevented a good retrieval of sequences solely based on this method. Some false positives, due to mis-annotation or too vague descriptions created marked noise. In contrast, the use of too specific annotations led to missing some sequences. For the 32 pathogenicity genes of our study, 5358 sequences were found by the keyword search; however, after analysis of the results, 86% of these sequences were identified as false positives.

Thus, at this moment, the only way to collect every sequence of a given gene is to combine keyword retrieval and a search by similarity (15). Keyword analysis often allows an estimation of the proportion of false positives and false negatives from a similarity method. False positives found by the similarity search provide sequences that can be used as outgroups in phylogenetic analyses or selectivity checks. They serve to verify efficiently if the published PCR primers are truly specific to the pathogenicity gene under study and do not also bind to other similar genes with a different function.

Sequences of each gene were then de-replicated: sequences contained into a longer sequence or identical sequences were removed in order to reduce the size of dataset, thus keeping only unique sequences. Unique sequences, corresponding to each target gene, were aligned with MUSCLE version 3.8.31 (23). Some outgroup sequences were kept to root phylogenetic trees, when possible (*i.e.* if they could be properly aligned). Each multiple sequence alignment was visually checked and corrected if necessary. Phylogenetic analyses were performed using a distance method (BIONJ (32)) and a maximum likelihood method (PhyML, version 3.0 (38)) using tools integrated into SeaView (37).

Gene names, protein names and annotations describing the sequences were analyzed. Using the species name or genus name, these annotations and specific keywords (such as PCR, primers, amplification, identification...), requests were made using Entrez at NCBI (PubMed), Jane (70) and eTBLAST (24) in order to retrieve a combined list of relevant PubMed IDentification numbers (PMID). Some requests yielded up to hundreds of publications. Each article was downloaded in PDF format and relevant short nucleic acid sequences were extracted from each file using regular expressions. Oligomers found at least once in the set of target sequences were selected for further analyses (Table S1).

The melting temperatures (Tm) of each primer were computed for each genetic variant of the target gene with the online software OHM (19) or a specific Python program; however, it should be noticed in our results that Tms returned by OHM are often slightly underestimated. OHM was mainly used in this study to check the coverage and the specificity of primers. Tms were confirmed either by dnaMATE (60) or a specific Python program. Primers with a Tm ranging from 55°C to 60°C for every target sequence were considered valid. Finally, the publication date of each primer was retrieved in order to follow the evolution of the proportion of valid and invalid primers over time. For primers cited in several articles, the earliest date was selected as the original publication date, and the difference between the earliest and the most recent date was used to estimate the duration of use or lifetime of a primer. These steps were repeated for each gene of the two pathogenicity islands.

Because different methods used to calculate a Tm can give different results, each Tm was computed using the basic (55) (bas), the salt-adjusted (42) (Sal) and three nearest-neighbor (6, 67, 73) (Bre, San and Sug) methods, with dnaMATE (60). In addition, the presence of hairpins and dimer formations was checked for each valid primer set using OligoAnalyzer 3.1 (http://eu.idtdna.com/analyzer/Applications/OligoAnalyzer/). Primers in a set that could hybridize with a free energy (ΔG) lower than −9 kcal/mole were removed.

From the alignment of every allele of a gene, conserved regions, of 18 bp or more and containing at most 2 ambiguities, were used to design primers. Then primers with a Tm ranging from 55°C to 60°C were selected. In parallel, primers were designed with two dedicated programs using a multiple alignment of sequences: Prifi (28) and Primaclade (31). These software programs have the advantages of being easily configurable and usable, since they are web applications with many parameters. Several parameters were refined: a minimum Tm of 55°C, a maximum Tm of 60°C, a minimum primer length of 18 bp, a maximum primer length of 40 bp and an interval of optimal primer length from 20 bp to 40 bp.

## Results

Every genetic variant of each gene and every relevant primer published in the scientific literature was retrieved using a semi-automated procedure. From 32 well-characterized pathogenicity genes, we found and analyzed 780 gene sequences and 230 different primers. We assessed the quality and specificity of each primer by comparison to each known allele of a target gene and related (similar) sequences. In this survey, we sought primers hybridizing to coding sequences (CDS) of a gene. Non-coding parts are less conserved than a CDS, and are likely to be less relevant for amplifying every gene variant.

The number of publicly available gene sequences was very variable, mostly depending upon the biological importance of the gene or its historical discovery (Table S1). In some cases (*e.g. ctxA* or *ctxB*), many sequences were found but corresponded to few unique alleles. This reflects, for these genes, the important effort of re-sequencing different strains, often resulting in identical sequences. Similarly, the number of primers was very variable (Table S1). Some pathogenicity genes, such as *acfA* or *acfC*, had only one published primer, although a minimum of two is required for PCR amplification. These results were seemingly caused by a design in non-coding regions (21), by the presence of an additional restriction site added to the primers (12) leading to the failure of our automated process, or finally when a larger genomic fragment was amplified with primers located within two different genes (59). In other cases, the number of primers was much higher (*e.g. ctxA*, *ctxB*, *zot*, *etc.*), for genes that had often been used in detection methods (20, 26, 72).

Surprisingly, only 32% of collected primers were valid for detection (predicted Tm ≥55°C), highlighting a problem in primer design even for newly published primers or the absence of a redesign of older primers when new gene sequences become available (Table S2). Using a Tm threshold of 50°C or no threshold showed few differences (Table S3). Curiously, *ctxB* and *tcpA* have several published primers, but no valid primer. Interestingly, this is a consequence of the high re-sequencing of these two genes, and the appearance of variant sequences with which old primers do not bind well. The discovery of new alleles therefore decreases the probability that a published primer remains valid (Table S4). For the *ctxB* gene, single nucleotide polymorphisms (SNPs) were observed along the sequences. Only 5 regions were identified with perfect identity between each *ctxB* sequence (AF463402, positions 1–31, 33–55, 139–164, 166–199, 344–72). Unfortunately, no published primer was designed in these areas. *tcpA* is a gene involved in the formation of a type IV *pilus* named TCP, leading to adhesion to the host. A functional TCP is needed for an immune response in humans (48). *tcpA* must adapt to the immune system, and due to this strong evolution pressure, sequences retrieved for the *tcpA* gene showed important diversity. *tcpA* nucleotide sequences share only 48.6% overall similarity, and only one region can be used for primer design (EU362122, positions 11–32). As for *ctxB*, no primer published for *tcpA* corresponded to this conserved domain, explaining the lack of valid primers for these two genes.

We were able to design pairs of primers for each of these genes (Table S5). In the difficult case of *tcpA*, the reverse primer had to be designed within the sequence of *tcpB*, a gene adjacent to *tcpA*. Both Prifi (28) and Primaclade (31) were used to design primers for *ctxB* and *ctxA*, a gene having valid published primers. While Primaclade retrieved several possible primers, PriFi returned only the four best couples. These two software programs provided different results for the same data. For *ctxB*, Primaclade provided primers with 1 or 2 ambiguities while Prifi created primers without ambiguity. The *ctxA* gene was chosen to test if these programs were able to generate all or part of the published primers. Because of the low number of results, Prifi retrieved only new primers for *ctxA*, whereas Primaclade retrieved 9 out of 19 valid published primers for *ctxA* (Table S5).

**Fig. 1:**
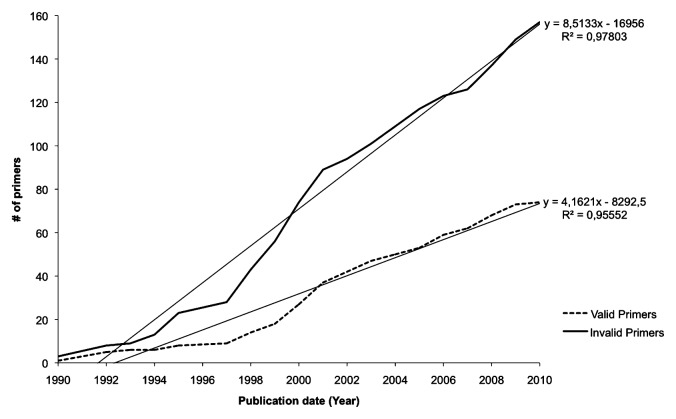
Cumulative numbers of valid and invalid published PCR primers used for amplification of *V. cholerae* pathogenicity genes. Numbers of primers are plotted as a function of their publication date. Dotted curve: invalid primers, full curve: valid primers. Straight lines are trend curves.

Publication dates of each primer were finally used to analyze if the first date of publication could be correlated with efficiency. Although the number of valid primers increased with time (α=4.2), invalid primers had a higher growth rate (α=8.5), showing an almost stable ratio of being twice more invalid than valid primers, independently of their publication date (Fig. 1); thus, unlike expectations, no significant improvement was observed over time, despite new bioinformatic tools being published almost every year (2, 4, 7, 9, 18, 19, 22, 28–31, 33, 34, 50, 57, 60, 62–64, 66, 74). Detailed information can be found in Table S10. Remarkably, the lifetime of a primer (Fig. S1), *i.e.* the number of years it is cited in the literature, showed that some invalid primers had been used for many years (for example, 6 invalid primers have been used for more than 15 years); by contrast, a large number of primers have been used a few years only. We also detected copy/paste errors in some articles. For example, in Sarkar *et al.*(2002)(68), the *ctxA* forward primer, as shown in Table 2 of this article, actually corresponds to a sequence in the *ace* gene. Even the reference provided (58) is wrong, since this primer is not cited in this article. The sequences of the two primers designed to amplify the *ace* gene are also wrong and are found neither in *ace* nor in the *ctxA* coding sequences. BLAST analysis showed that these primers were found 139 bp before a predicted DNA-binding protein of *Bacteroides xylanisolvens* and 241 bp before *ace* in *V. cholerae*, respectively. In another article describing the presence of *V. cholerae* in mussels following an outbreak in Denmark and Sweden (17), *ctxA* genes of *V. cholerae* were not detected by PCR, while biochemical tests identified the presence of the gene product, which is likely due to an inappropriate reverse primer from Brasher *et al.*(5). These two results strongly suggest that more in-depth analyses of primers should be performed before proceeding to molecular detection; however, it is a difficult task for biologists without programming ability and we hope that this study will help them in selecting proper primers.

In some cases, invalid published primers could be modified in order to obtain perfect sensitivity for each genetic variant of the gene. Their improvements simply consisted in adding at most 2 ambiguities, as shown in Tables S6, S7 and S8. Applied to the whole dataset, such a procedure could possibly restore the detection capability of 37.7% of invalid primers.

## Discussion

Our survey clearly revealed that two thirds of published primers are not able to properly detect every genetic variant of a gene. Moreover, design did not improve with time, despite major advances in primer design over the years. Their lifetime, *i.e.* the number of times they are cited in the literature, is also not a factor allowing the selection of good primers. Note that we were not able to retrieve all publications that had used a given primer, because we used automated regular expression to extract oligomer sequences from articles. Publications that refer to a given primer using a citation to a previous work, without providing the sequence of that primer, were not identified by our procedure. Surprisingly, the two genes with the most published primers, *ctxB* and *tcpA*, do not have any valid primer. Improvements of these primers by adding ambiguities could theoretically restore 11 primers in *ctxB* and 4 in *tcpA* (Tables S6 and S7). Nevertheless, because of its high evolutionary rate, the results are probably not definitive for *tcpA*. The identification of conserved regions between every genetic variant is of course important in the design of universal primers but, for genes with a high mutation rate, the use of ambiguities is required.

However, it should be noted that the estimated Tm used to determine valid primers was arbitrary fixed from 55°C, according to handbooks of molecular biology and since the difference with no threshold or a threshold of 50°C was weak (Table S3). The computation of theoretical Tm should be used with caution, since each estimation method may return different results; some primers actually work experimentally even with a theoretical Tm below 55°C. Thus, the critical information used in this study to determine the validity of a primer is its specificity and its coverage.

Our study thus reflects two problems. First, primers designed 5 to 10 years ago are currently used, and usually have not been reassessed using new sequences present in the latest release of public databases, in order to check their efficiency and improve them if necessary (or design new primers). Second, some recent primers are invalid, showing that the primers were not designed correctly, despite the availability of numerous tools for primer design.

One problem lies in the selection of a given tool to design or check the validity of primers. Some tools only check primer’s thermodynamic properties, such as hairpin formation, dimers of primers or Tm. NetPrimer (http://www.premierbiosoft.com/netprimer/index.html) or OligoCalc (50) can analyze one primer at a time, while dnaMATE (60) or OHM (19) can assess a list of primers. OHM was specifically designed to compute Tm of primers against several target and non-target sequences. An interesting feature is the ease of visualizing how primers amplify sequences, either as a picture or used with Treedyn (11) to annotate phylogenetic trees composed of target and non-target sequences. With a color code, the specificity and the sensitivity of primers can be easily estimated by eye. To our knowledge, only two software progams have the ability to assess the thermodynamic properties of degenerated primers: OligoAnalyzer (http://eu.idtdna.com/analyzer/Applications/OligoAnalyzer/) and dPrimer (10).

The most popular tool to design primers is perhaps Primer3 (64), available either stand-alone or as a web server. Similar programs and more information on the characteristics of design primer software can be found in Table S10. The NCBI website now proposes Primer-BLAST (http://www.ncbi.nlm.nih.gov/tools/primer-blast/), which allows the specificity of newly designed primers to be checked, but does not take into account genetic variations present in a gene. In conclusion, the software cited above is not really relevant or easy to use when primers must be designed in order to target every genetic variant of a gene, and not a single sequence. This observation could also explain the fact that our survey revealed a majority of invalid published primers, since primers were probably designed using only one target sequence. Few tools can deal with several sequences to generate primers (*e.g.* PriFi (28), Primaclade (31) or PrimerHunter (22)). These programs, using multiple alignments of sequences, can produce degenerated PCR primers, which are required when gene sequences carry intrinsic variations such as SNPs or deletions.

Finally, one cause of badly designed primers is the difficulty in specifically retrieving every genetic variant of a gene. Generally, BLAST searches are used to perform this task; however in many cases, a given gene is present within a larger genomic fragment and it is tedious to manually retrieve and extract every gene sequence. Also, when a gene has a high rate of mutation, the BLAST results might be difficult to read. Finallym these investigations must be performed after each release of the public database. By collecting every gene allele and every published PCR primer we were able to assess most of the published primers and to propose possible improvements. We showed that adding ambiguities can improve the efficiency of many published primers, or that increasing their length could increase their Tm. Strains carrying an atypical or a rare gene variant would thus now be detected.

Failure of amplification due to the bad choice of a primer set will probably not show up when the primers are used to amplify DNA purified from a culture. In such cases, there is relatively little non-target DNA and amplification might succeed despite mismatches between a primer and a gene sequence. This could be quite different if amplification is used to assess the presence of a pathogen in environmental samples. In such a case, a large abundance of “foreign” DNA would give rise to detrimental thermodynamic conditions, and likely lead to a failure of the detection system, despite the presence of a pathogen. This is why we suggest that procedures to detect genes by PCR amplification should always be tested using not only DNA from cultured strains but also with the addition of DNA extracted from the environment.

In order to document this problem, we analyzed the primers used in a recent article (71) where a series of PCR amplification targeted pathogenicity genes to detect variants of *V. cholerae* in the digestive tracts of 14 fish species. As shown by our analyses (Fig. S2), some of the primers used were not optimal, and the presence or absence of potential virulence genes detected could have been biased by a failure of PCR amplification. In particular all strains were found to be negative for *tcpA*, but the primers used were far from optimal (Fig. S2-G). The horizontal transfer of virulence genes between *V. cholerae* and closely related species, recently described for *V. mimicus*(77), can explain the lack of specificity of some primers. We provide the complete list of gene sequences (format fasta) and primers at www.patho-genes.org/Project_cholera.html.

In conclusion, virulence genes are dispersed among environmental strains of *V. cholerae* belonging to diverse serogroups, which constitute an environmental reservoir of virulence genes (25). The origin of new epidemic strains from the environment is likely since the different virulence-associated genes are scattered among environmental vibrios, which possess lower virulence potential than the epidemic strains. Some particular ecological setting may favor increased genetic exchange among strains, thus promoting multiple-gene transfers needed to assemble the critical combination of genes required for pandemic spread (26). A reference database of gene sequences and primers to amplify them might be useful in order to survey such processes and understand which factors may promote the rise of a new virulent strain.

## Supplementary Material



## Figures and Tables

**Table 1:** List of valid primer sets. From valid primers, a list of valid primer sets was generated that can be used to detect every allele of their target gene specifically. Dimer formations were checked. Tms were calculated as described in the methods, and the Tms predicted for use of each set are indicated

		Valid Primer Set		Tm (°C)					Amplicon Size (pb)
		
	Gene	Foward	Reverse	Bas	Sal	Bre	San	Sug	
CTX Prophage	*ace*	CCGCTTATCCAACAGGCTATC	AGGTTTAACGCTCGCAGGGCG	49.5	54.8	59.8	49.2	52.8	133

	*cep*	GGCTTAATTCGTAAGGCTAAA	AAACAGCAAGAAAACCCCGAGT	48.5	55.5	54.7	44.8	50.4	195

	*ctxa*	CTCAGACGGGATTTGTTAGGCACG	TATGCCCCTAATACATCATTAACG	52.3	60.1	58.3	47.2	52.8	168
		CTCAGACGGGATTTGTTAGGCACG	TCTATCTCTGTAGCCCCTATTACG	55.7	63.5	57.2	49.4	55.9	301
		ATGATCATGCAAGAGGAACTC	TATGCCCCTAATACATCATTAACG	50.4	57.4	55.6	46.7	51.5	186
		ATGATCATGCAAGAGGAACTC	TCTATCTCTGTAGCCCCTATTACG	50.4	57.4	55.6	46.7	51.5	319
		TTTGTTAGGCACGATGATGGAT	TATGCCCCTAATACATCATTAACG	51.1	58.4	60.5	49.1	53.2	157
		TTTGTTAGGCACGATGATGGAT	TCTATCTCTGTAGCCCCTATTACG	51.1	58.4	60.5	49.1	53.2	290
		GGCAGATTCTAGACCTCCTGATGAAATAAA	CGTGCCTAACAAATCCCGTCTGAG	58.9	68.0	65.6	53.3	59.7	145
		GGCAGATTCTAGACCTCCTGATGAAATAAA	TATGCCCCTAATACATCATTAACG	52.3	60.1	58.3	47.2	52.8	290
		GGCAGATTCTAGACCTCCTGATGAAATAAA	ATCCATCATCGTGCCTAACAAA	51.1	58.4	60.5	49.1	53.2	154
		GGCAGATTCTAGACCTCCTGATGAAATAAA	TCTATCTCTGTAGCCCCTATTACG	55.7	63.5	57.2	49.4	55.9	423
		GGCAGATTCTAGACCTCCTGATGAAATAAA	CCCGTCTGAGTTCCTCTTGC	55.9	62.5	61.1	51.4	55.3	131
		GGCAGATTCTAGACCTCCTGATGAAATAAA	GGGCACTTCTCAAACTAATTGAGGTGGAAACA	58.9	68.0	65.6	53.3	59.7	187
		GGCAGATTCTAGACCTCCTGATGAAATAAA	TGAGTTCCTCTTGCATGATCA	50.5	57.4	58.2	48.2	52.7	125
		GCAAGAGGAACTCAGACGGG	TATGCCCCTAATACATCATTAACG	52.3	60.1	58.3	47.2	52.8	178
		GCAAGAGGAACTCAGACGGG	TCTATCTCTGTAGCCCCTATTACG	55.7	63.5	57.2	49.4	55.9	311
		TGTTTCCACCTCAATTAGTTTGAGAAGTGCCC TATGCCCCTAATACATCATTAACG		52.3	60.1	58.3	47.2	52.8	134
		TGTTTCCACCTCAATTAGTTTGAGAAGTGCCC TCTATCTCTGTAGCCCCTATTACG		55.7	63.5	57.2	49.4	55.9	267
		TGATCATGCAAGAGGAACTCA	TATGCCCCTAATACATCATTAACG	50.5	57.4	58.2	48.2	52.7	185
		TGATCATGCAAGAGGAACTCA	TCTATCTCTGTAGCCCCTATTACG	50.5	57.4	58.2	48.2	52.7	318
		AGTCAGGTGGTCTTATGCC	CGTGCCTAACAAATCCCGTCTGAG	51.1	57.3	53.8	47.8	50.3	113
		AGTCAGGTGGTCTTATGCC	TATGCCCCTAATACATCATTAACG	51.1	57.3	53.8	47.8	50.3	258
		AGTCAGGTGGTCTTATGCC	ATCCATCATCGTGCCTAACAAA	51.1	57.3	53.8	47.8	50.3	122
		AGTCAGGTGGTCTTATGCC	TCTATCTCTGTAGCCCCTATTACG	51.1	57.3	53.8	47.8	50.3	391
		AGTCAGGTGGTCTTATGCC	GGGCACTTCTCAAACTAATTGAGGTGGAAACA	51.1	57.3	53.8	47.8	50.3	155
		AACTCAGACGGGATTTGTTAGG	TATGCCCCTAATACATCATTAACG	52.3	60.1	58.3	47.2	52.8	170
		AACTCAGACGGGATTTGTTAGG	TCTATCTCTGTAGCCCCTATTACG	53.0	60.3	58.5	49.0	53.2	303

	*ctxb*	TCGTATACAGAATCTCTAGCTGGAAA	GCCATACTAATTGCGGCAATCGC	54.8	63.1	58.9	50.0	56.9	229

	*orfu*	CGTCACACCAGTTACTTTTCG	CCTAAACAAAATGAGCATGGC	50.5	57.4	58.5	46.9	51.5	1096
		CGTCACACCAGTTACTTTTCG	GCGTGAAACTTCGTATTGAGCT	52.4	59.4	57.2	48.0	52.8	414
		CGTCACACCAGTTACTTTTCG	CAATAAGGATAAATGCAGCGCTCTG	52.4	59.4	57.2	48.0	52.8	237
		ATGCGCTATTTTCTACTGTTTTTG	CGAAAAGTAACTGGTGTGACG	50.6	58.4	58.0	47.3	53.8	108
		ATGCGCTATTTTCTACTGTTTTTG	CCTAAACAAAATGAGCATGGC	50.5	57.4	58.5	46.9	51.5	1184
		ATGCGCTATTTTCTACTGTTTTTG	CATGCAGCCATCAAATAACACC	50.6	58.4	58.0	47.3	53.8	155
		ATGCGCTATTTTCTACTGTTTTTG	GCGTGAAACTTCGTATTGAGCT	50.6	58.4	58.0	47.3	53.8	523
		GGTGTTATTTGATGGCTGCATG	CCTAAACAAAATGAGCATGGC	50.5	57.4	58.5	46.9	51.5	1050
		GGTGTTATTTGATGGCTGCATG	GCGTGAAACTTCGTATTGAGCT	53.0	60.3	61.4	49.3	53.5	389
		GGTGTTATTTGATGGCTGCATG	CAATAAGGATAAATGCAGCGCTCTG	53.0	60.3	61.4	49.3	53.5	191
		AGCTCAATACGAAGTTTCACGC	CCTAAACAAAATGAGCATGGC	50.5	57.4	58.5	46.9	51.5	682
		CAGAGCGCTGCATTTATCCTTATTG	CCTAAACAAAATGAGCATGGC	50.5	57.4	58.5	46.9	51.5	883
		CAGAGCGCTGCATTTATCCTTATTG	GCGTGAAACTTCGTATTGAGCT	53.0	60.3	59.8	50.2	55.7	231
		AGAGCGCTGCATTTATCCTTATTG	CCTAAACAAAATGAGCATGGC	50.5	57.4	58.5	46.9	51.5	882
		AGAGCGCTGCATTTATCCTTATTG	GCGTGAAACTTCGTATTGAGCT	53.0	60.3	59.8	50.2	55.7	230

	*zot*	GCCACTTTAACCGCGCCAC	CGATAACGCTCATCACCAACAGTG	55.4	61.6	64.9	53.5	55.9	450
		GCCACTTTAACCGCGCCAC	CAAAGCCGACCAATACAAAAACCAA	54.4	62.5	65.8	51.8	55.9	408
		CGGCGCTGTGGAAAGACAG	CGATAACGCTCATCACCAACAGTG	55.4	61.6	64.2	52.5	57.1	267
		TCGCTTAACGATGGCGCGTTTT	CAAAGCCGACCAATACAAAAACCAA	54.8	62.1	68.6	54.8	59.8	677
		TCGCTTAACGATGGCGCGTTTT	GTTAGGCGTGGTTAGGCAGATATC	54.8	62.1	68.6	54.8	59.8	219
		GATATCTGCCTAACCACGCCTAAC	CGGCGCTGTGGAAAGACAG	55.4	61.6	64.2	52.5	57.1	274
		GATATCTGCCTAACCACGCCTAAC	CACTGTTGGTGATGAGCGTTATCG	57.4	65.2	64.9	52.7	58.3	523
		GATATCTGCCTAACCACGCCTAAC	TTGGTTTTTGTATTGGTCGGCTTTG	54.4	62.5	65.8	51.8	55.9	481

TCP Prophage	*acfb*	TTTGTCTGAGCCGTATGTCG	GAGCGTGCTTTATCATGGTCGAT	51.8	58.4	58.7	48.8	53.7	377
		TTTGTCTGAGCCGTATGTCG	CAGCAACCACAGCAAAACC	51.1	57.3	59.1	49.0	51.6	1066
		ATCGACCATGATAAAGCACGCTC	CAGCAACCACAGCAAAACC	51.1	57.3	59.1	49.0	51.6	711

	*alda*	GTCAATGGATGAAGCCACACAGTG	GGTACAAACCTCACCTTGGTT	52.4	59.4	56.9	49.2	50.8	832

	*int*	GAAGTAATGAAACCGATAAGTGG	TGCTTTGTACCAGTCACAGATAG	51.7	59.3	55.9	46.0	51.2	346

	*tcpf*	GAGTTCCACATGCAGAAACAGGA	TCTCTGAATATGCTTTGCTATACAGT	53.2	61.6	57.0	49.0	56.0	239
		GAGTTCCACATGCAGAAACAGGA	CACACCACTTCCATCTCCT	51.1	57.3	54.6	47.7	50.3	211
		GACGCATACCCATCGACAGA	TCTCTGAATATGCTTTGCTATACAGT	53.2	61.6	57.0	49.0	56.0	765
		GACGCATACCCATCGACAGA	TCCTGTTTCTGCATGTGGAACTC	53.8	60.5	60.8	50.6	54.3	548
		GACGCATACCCATCGACAGA	AACAGGGTCATAGATAACTCC	50.4	57.4	51.3	45.3	49.1	566
		GACGCATACCCATCGACAGA	CACACCACTTCCATCTCCT	51.1	57.3	54.6	47.7	50.3	737
		GGAGTTATCTATGACCCTGTT	TCTCTGAATATGCTTTGCTATACAGT	50.4	57.4	51.3	45.3	49.1	219
		GGAGTTATCTATGACCCTGTT	CACACCACTTCCATCTCCT	50.4	57.4	51.3	45.3	49.1	191

	*tcpi*	TAACGAGCTCGACACTATTGCC	TGCCTGCTGAGAACTAAGGCTA	54.8	62.1	60.5	52.4	57.7	861
		TAACGAGCTCGACACTATTGCC	CGACTGCTTTATCGCGAAGT	51.8	58.4	59.4	49.4	55.7	756
		TAGCCTTAGTTCTCAGCAGGCA	CGACTGCTTTATCGCGAAGT	51.8	58.4	59.4	49.4	55.7	124
		CGACTGCTTTATCGCGAAGT	CCTGCGTTCTTTTATCTGACCATC	51.8	58.4	59.4	49.4	55.7	720

	*tcpq*	ACCGTGTAAATCAGCCCAAG	AGCCAACTCAGTTAAAACTTGTTC	51.8	58.4	58.8	49.5	53.3	112
		GCACAAGGAGAGATGCACAA	CTTGGGCTGATTTACACGGT	51.8	58.4	58.8	49.5	53.3	215
		GCACAAGGAGAGATGCACAA	AGCCAACTCAGTTAAAACTTGTTC	51.8	58.4	58.8	49.5	53.3	308

	*toxt*	TACGCGTAATTGGCGTTGGGCAG	CTTGGTGCTACATTCATGG	48.9	55.2	53.7	44.7	48.9	245
		TGGGCAGATATTTGTGGTGA	CTTGGTGCTACATTCATGG	48.9	55.2	53.7	44.7	48.9	229
