# Supplementary material for: In Silico Analyses of Primers Used to Detect the Pathogenicity Genes of Vibrio cholerae
Source: Microbes Environ. 2012 May 17;27(3):250–6. doi: 10.1264/jsme2.ME11317 (PMC4036039; doi:10.1264/jsme2.ME11317)
Supplement: Supplementary file 1 [file 27_250_s1.pdf]

Fig. S1: Lifetime of valid and invalid published PCR primers used to amplify *V. cholerae* pathogenicity genes. For each primer, total number of primers is plotted against the number of years it was still cited in the literature after its first publication. Dotted curve: invalid primers, full curve: valid primers (124 invalid primers and 57 valid primers with a 1-year lifetime are not shown). Note the large number of primers used one or two years only (5 valid and 4 invalid primers).

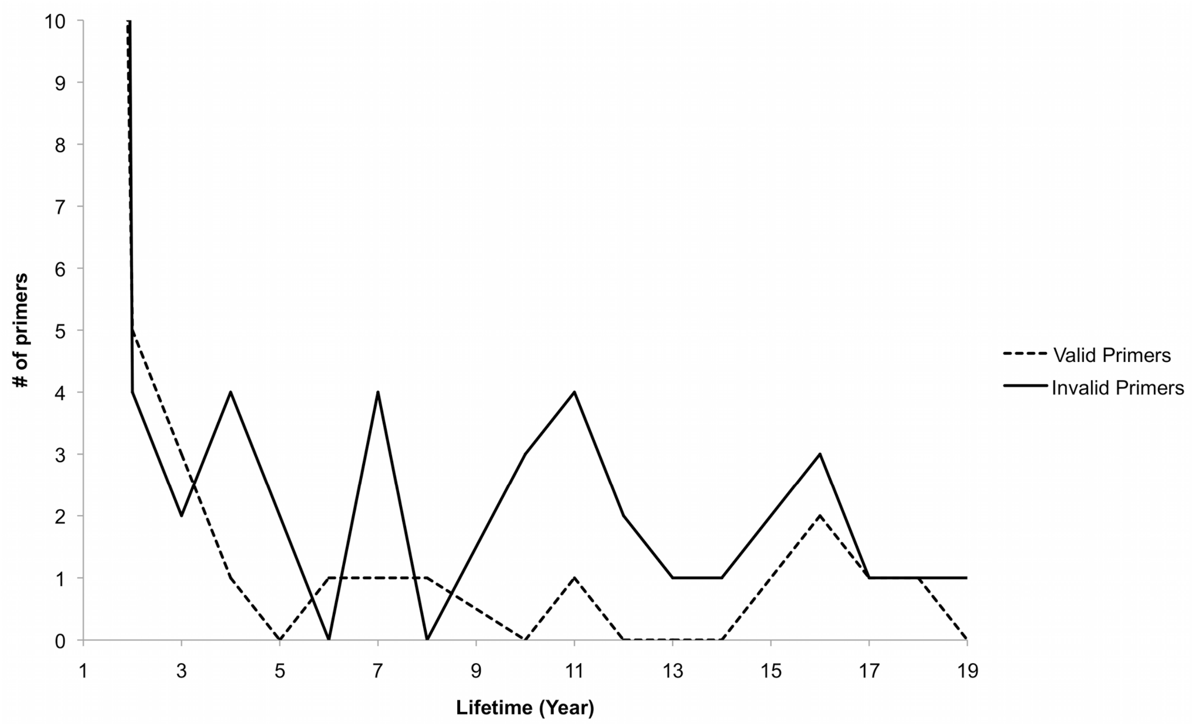

Fig. S2: Efficiency of PCR primers used to amplify genes of *V. cholerae* in Senderovich et al. 2010. A phylogenetic analysis was performed with every genetic variant of *ctxA* (A), *hapA* (B), *hlyA* (C), *ompU* (D), *ompW* (E), *sto* (F), *tcpA* (G), *tcpI* (H), *toxR* (I), *zot* (J) genes. Tms were computed for each variant gene sequence and each primer using OHM. Accession number, number of other sequences identical to or contained in the sequence used in this tree, species names, and Tms are indicated (forward and reverse primers in columns from left to right). For strain, biovar and serotype information, see Table S9. Tms are symbolized by a color code described in the box on the right. A predicted Tm lower than the lowest temperature mentioned in the box is symbolized by a grey square. White boxes when a partial sequence does not encompass a primer. Please note that OHM tends to underestimate the Tm of primers (as compared to other tools).

ctxA: the forward and reverse primers amplify every allele of the gene and are specific of *V. cholerae*.

hapA: the forward and reverse primers amplify every allele of the gene and are specific of *V. cholerae*. However, a slight variation of Tm is observed for the reverse primer and the sequence CP001236, and a lower Tm (50.8°C) should be used for the detection.

hlyA: the forward and reverse primers detect every known sequence of *V. harveyi* but cannot amplify every allele of the gene in *V. cholerae* (AF194418, D58374, AY427780).

ompU: the forward and reverse primers amplify every allele of the gene in *V. cholerae*, but also detect some sequences of *V. mimicus* (e.g. DQ356331).

ompW: the high variation of Tms (from 46 to 54°C) for the second couple is probably too large for a proper detection. Only the first couple is probably useful to amplify *ompW* in *V. cholerae*.

sto: the couple of primer amplifies only one allele (M85198) of the gene in *V. cholerae*.

tcpA: the reverse primer is the primer targeting the classical strain of *V. cholerae*. The one targeting the El tor strain was not studied because of its localization outside the CDS. As shown in this figure, the forward and reverse primers cannot amplify every allele of the gene in *V. cholerae*, but will detect two sequences of *V. mimicus* (AF315787, DQ356010). The Tms of the forward primer vary from 43.8 to 51.4°C, according to the method of computation of Tm, with a high Tm (51.4°C) for the sequences of *V. mimicus*. For the reverse primers, the Tms vary from 52.1 to 52.5°C. The efficiency of this primer cannot be appreciated for sequences of *V. mimicus* because only partial sequences are available for the target region.

tcpI: the forward and reverse primers amplify every allele of the gene.

toxR: the primers of the first (Nandi et al. 2000) and the second (Halpern et al. 2005) published couples amplify every allele of the gene in *V. cholerae*. However, a variation of Tm is observed for the foward primer of the second couple for sequence CP001485. The lowest Tm (45.8°C) should be used for the detection, but this condition can favor the amplification of sequences of *V. harveyi*. Indeed, because of the partial sequences, it is not possible to estimate the efficiency of couples for the outgroup sequences, and therefore their specificity toward *V. cholerae*. As a result, *toxR* is not a validated genetic marker for the detection of *V. cholerae*.

zot: the forward and the reverse primers amplify every allele of the gene in *V. cholerae*. A variation of Tm is observed for the foward primer and sequence AF220606. A low Tm (47.7°C) should be used for the detection. The specificity of this couple cannot be estimated because of the partial sequence of *V. mimicus*. The *zot* gene is therefore not validated for detecting *V. cholerae*.

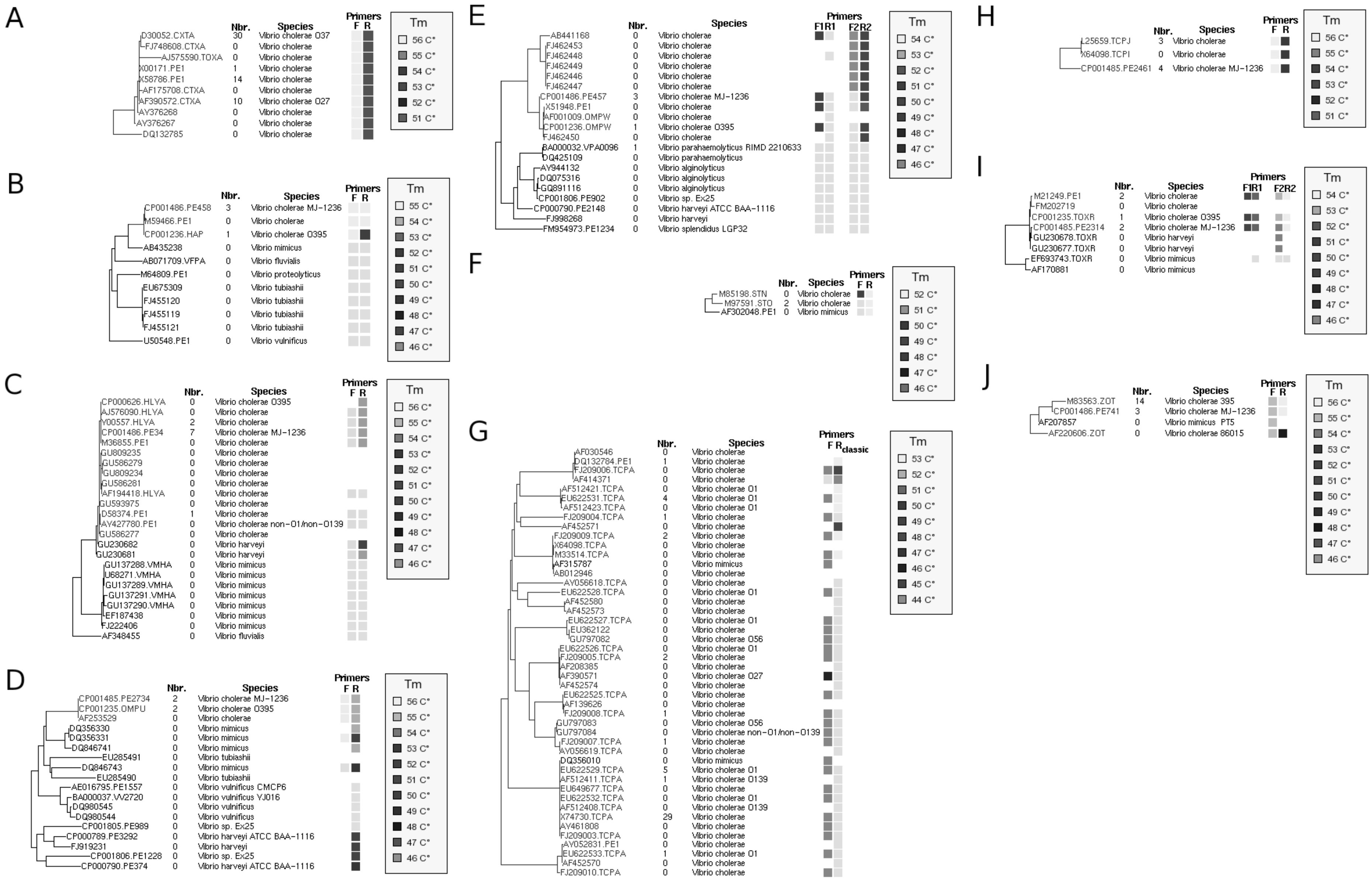

A
Nbr.
Species
Primers
F R
Tm
56 C°
55 C°
54 C°
53 C°
52 C°
51 C°
D30052.CXTA 30 Vibrio cholerae O37
FJ748608.CTXA 0 Vibrio cholerae
AJ575590.TOXA 0 Vibrio cholerae
X00171.PE1 1 Vibrio cholerae
X58786.PE1 14 Vibrio cholerae
AF175708.CTXA 0 Vibrio cholerae
AF390572.CTXA 10 Vibrio cholerae O27
AY376268 0 Vibrio cholerae
AY376267 0 Vibrio cholerae
DQ132785 0 Vibrio cholerae
B
Nbr.
Species
Primers
F R
Tm
55 C°
54 C°
53 C°
52 C°
51 C°
50 C°
49 C°
48 C°
47 C°
46 C°
CP001486.PE458 3 Vibrio cholerae MJ-1236
M59466.PE1 0 Vibrio cholerae
CP001236.HAP 1 Vibrio cholerae O395
AB435238 0 Vibrio mimicus
AB071709.VFPA 0 Vibrio fluvialis
M64809.PE1 0 Vibrio proteolyticus
EU675309 0 Vibrio tubiashii
FJ455120 0 Vibrio tubiashii
FJ455119 0 Vibrio tubiashii
FJ455121 0 Vibrio tubiashii
U50548.PE1 0 Vibrio vulnificus
C
Nbr.
Species
Primers
F R
Tm
56 C°
55 C°
54 C°
53 C°
52 C°
51 C°
50 C°
49 C°
48 C°
47 C°
46 C°
CP000626.HLYA 0 Vibrio cholerae O395
AJ576090.HLYA 0 Vibrio cholerae
Y00557.HLYA 2 Vibrio cholerae
CP001486.PE34 7 Vibrio cholerae MJ-1236
M36855.PE1 0 Vibrio cholerae
GU809235 0 Vibrio cholerae
GU586279 0 Vibrio cholerae
GU809234 0 Vibrio cholerae
GU586281 0 Vibrio cholerae
AF194418.HLYA 0 Vibrio cholerae
GU593975 0 Vibrio cholerae
D58374.PE1 1 Vibrio cholerae
AY427780.PE1 0 Vibrio cholerae non-O1/non-O139
GU586277 0 Vibrio cholerae
GU230682 0 Vibrio harveyi
GU230681 0 Vibrio harveyi
GU137288.VMHA 0 Vibrio mimicus
U68271.VMHA 0 Vibrio mimicus
GU137289.VMHA 0 Vibrio mimicus
GU137291.VMHA 0 Vibrio mimicus
GU137290.VMHA 0 Vibrio mimicus
EF187438 0 Vibrio mimicus
FJ222406 0 Vibrio mimicus
AF348455 0 Vibrio fluvialis
D
Nbr.
Species
Primers
F R
Tm
56 C°
55 C°
54 C°
53 C°
52 C°
51 C°
50 C°
49 C°
48 C°
47 C°
46 C°
CP001485.PE2734 2 Vibrio cholerae MJ-1236
CP001235.OMPU 2 Vibrio cholerae O395
AF253529 0 Vibrio cholerae
DQ356330 0 Vibrio mimicus
DQ356331 0 Vibrio mimicus
DQ846741 0 Vibrio mimicus
EU285491 0 Vibrio tubiashii
DQ846743 0 Vibrio mimicus
EU285490 0 Vibrio tubiashii
AE016795.PE1557 0 Vibrio vulnificus CMCP6
BA000037.VV2720 0 Vibrio vulnificus YJ016
DQ980545 0 Vibrio vulnificus
DQ980544 0 Vibrio vulnificus
CP001805.PE989 0 Vibrio sp. Ex25
CP000789.PE3292 0 Vibrio harveyi ATCC BAA-1116
FJ919231 0 Vibrio harveyi
CP001806.PE1228 0 Vibrio sp. Ex25
CP000790.PE374 0 Vibrio harveyi ATCC BAA-1116
E
Nbr.
Species
Primers
F1R1
F2R2
Tm
54 C°
53 C°
52 C°
51 C°
50 C°
49 C°
48 C°
47 C°
46 C°
AB441168 0 Vibrio cholerae
FJ462453 0 Vibrio cholerae
FJ462448 0 Vibrio cholerae
FJ462449 0 Vibrio cholerae
FJ462446 0 Vibrio cholerae
FJ462447 0 Vibrio cholerae
CP001486.PE457 3 Vibrio cholerae MJ-1236
X51948.PE1 0 Vibrio cholerae
AF001009.OMPW 0 Vibrio cholerae
CP001236.OMPW 1 Vibrio cholerae O395
FJ462450 0 Vibrio cholerae
BA000032.VPA0096 1 Vibrio parahaemolyticus RIMD 2210633
DQ425109 0 Vibrio parahaemolyticus
AY944132 0 Vibrio alginolyticus
DQ075316 0 Vibrio alginolyticus
GQ891116 0 Vibrio alginolyticus
CP001806.PE902 0 Vibrio sp. Ex25
CP000790.PE2148 0 Vibrio harveyi ATCC BAA-1116
FJ998268 0 Vibrio harveyi
FM954973.PE1234 0 Vibrio splendidus LGP32
F
Nbr.
Species
Primers
F R
Tm
52 C°
51 C°
50 C°
49 C°
48 C°
47 C°
46 C°
M85198.STN 0 Vibrio cholerae
M97591.STO 2 Vibrio cholerae
AF302048.PE1 0 Vibrio mimicus
G
Nbr.
Species
Primers
F R classic
Tm
53 C°
52 C°
51 C°
50 C°
49 C°
48 C°
47 C°
46 C°
45 C°
44 C°
AF030546 0 Vibrio cholerae
DQ132784.PE1 1 Vibrio cholerae
FJ209006.TCPA 0 Vibrio cholerae
AF414371 0 Vibrio cholerae
AF512421.TCPA 0 Vibrio cholerae O1
EU622531.TCPA 4 Vibrio cholerae O1
AF512423.TCPA 0 Vibrio cholerae O1
FJ209004.TCPA 1 Vibrio cholerae
AF452571 0 Vibrio cholerae
FJ209009.TCPA 2 Vibrio cholerae
X64098.TCPA 0 Vibrio cholerae
M33514.TCPA 0 Vibrio cholerae
AF315787 0 Vibrio mimicus
AB012946 0 Vibrio cholerae
AY056618.TCPA 0 Vibrio cholerae
EU622528.TCPA 0 Vibrio cholerae O1
AF452580 0 Vibrio cholerae
AF452573 0 Vibrio cholerae
EU622527.TCPA 0 Vibrio cholerae O1
EU362122 0 Vibrio cholerae
GU797082 0 Vibrio cholerae O56
EU622526.TCPA 0 Vibrio cholerae O1
FJ209005.TCPA 2 Vibrio cholerae
AF208385 0 Vibrio cholerae
AF390571 0 Vibrio cholerae O27
AF452574 0 Vibrio cholerae
EU622525.TCPA 0 Vibrio cholerae
AF139626 0 Vibrio cholerae
FJ209008.TCPA 1 Vibrio cholerae
GU797083 0 Vibrio cholerae O56
GU797084 0 Vibrio cholerae non-O1/non-O139
FJ209007.TCPA 1 Vibrio cholerae
AY056619.TCPA 0 Vibrio cholerae
DQ356010 0 Vibrio mimicus
EU622529.TCPA 5 Vibrio cholerae O1
AF512411.TCPA 1 Vibrio cholerae O139
EU649677.TCPA 0 Vibrio cholerae
EU622532.TCPA 0 Vibrio cholerae O1
AF512408.TCPA 0 Vibrio cholerae O139
X74730.TCPA 29 Vibrio cholerae
AY461808 0 Vibrio cholerae
FJ209003.TCPA 0 Vibrio cholerae
AY052831.PE1 0 Vibrio cholerae
EU622533.TCPA 1 Vibrio cholerae O1
AF452570 0 Vibrio cholerae
FJ209010.TCPA 0 Vibrio cholerae
H
Nbr.
Species
Primers
F R
Tm
56 C°
55 C°
54 C°
53 C°
52 C°
51 C°
L25659.TCPJ 3 Vibrio cholerae
X64098.TCPI 0 Vibrio cholerae
CP001485.PE2461 4 Vibrio cholerae MJ-1236
I
Nbr.
Species
Primers
F1R1
F2R2
Tm
54 C°
53 C°
52 C°
51 C°
50 C°
49 C°
48 C°
47 C°
46 C°
M21249.PE1 2 Vibrio cholerae
FM202719 0 Vibrio cholerae
CP001235.TOXR 1 Vibrio cholerae O395
CP001485.PE2314 2 Vibrio cholerae MJ-1236
GU230678.TOXR 0 Vibrio harveyi
GU230677.TOXR 0 Vibrio harveyi
EF693743.TOXR 0 Vibrio mimicus
AF170881 0 Vibrio mimicus
J
Nbr.
Species
Primers
F R
Tm
56 C°
55 C°
54 C°
53 C°
52 C°
51 C°
50 C°
49 C°
48 C°
47 C°
46 C°
M83563.ZOT 14 Vibrio cholerae 395
CP001486.PE741 3 Vibrio cholerae MJ-1236
AF207857 0 Vibrio mimicus PT5
AF220606.ZOT 0 Vibrio cholerae 86015

Table S1: Summary of results obtained from the analysis of the complete list of sequences retrieved for *V. cholerae* pathogenicity genes.

Columns 1: pathogenicity island, 2: most frequently used gene name, 3: total number of sequences retrieved, 4: total number of unique sequences, 5: number of alternate gene and protein names found, 6: published and 7: valid primers retrieved from the literature.

	Gene	Sequences	Unique Sequences	Keywords	Primers	Valid Primers
CTX Prophage	ace	9	3	2	8	4
	cep	7	3	2	3	2
	ctxA	65	10	12	41	18
	ctxB	205	24	17	23	0
	orfU	9	3	1	9	7
	zot	21	4	4	24	8
	rstA	29	16	8	8	0
	rstB	12	4	5	3	0
	rstR	12	2	4	3	2
TCP Prophage	acfA	19	7	3	0	0
	acfB	10	4	3	6	4
	acfC	9	1	2	1	1
	tagE	18	7	4	0	0
	aldA	30	14	7	6	2
	int	13	4	6	5	4
	tagA	18	10	4	4	0
	tagD	8	2	4	1	1
	tcpA	98	46	19	27	0
	tcpB	40	11	3	3	0
	tcpC	9	3	4	1	0
	tcpD	7	1	3	1	1
	tcpE	8	1	3	1	1
	tcpF	10	5	4	5	5
	tcpH	17	10	3	6	0
	tcpI	10	3	5	7	4
	tcpJ	10	4	5	4	0
	tcpP	21	9	3	12	2
	tcpQ	17	8	3	5	3
	tcpR	8	1	3	0	0
	tcpS	8	1	3	0	0
	tcpT	8	2	3	0	0
	toxT	15	7	7	13	4
**Sum**		780	230	159	230	73

Table S2: list of valid published primers.

Tms were calculated as described in the methods.

See also http://protein.bio.puc.cl/cardex/servers/dnaMATE/index.html for more details.

	Gene	Valid Primer	Tm (°C)				
			Bas	Sal	Bre	San	Sug
CTX Prophage	ace	CCGCTTATCCAACAGGCTATC	54.4	61.3	59.9	49.5	54.6
		CCGCTTATCCAACAGGCTAT	51.8	58.4	58.1	48.6	53.3
		TTTAACGCTCGCAGGGC	49.5	54.8	59.8	49.2	52.8
		CGCCCTGCGAGCGTTAAACCT	55.9	62.5	64.8	54.5	57.8
	cep	TTTAGCCTTACGAATTAAGCC	48.5	55.5	54.7	44.8	50.4
		AAACAGCAAGAAAACCCCGAGT	53.0	60.3	62.3	52.1	55.0
	ctxa	CTCAGACGGGATTTGTTAGGCACG	59.1	66.9	66.9	54.3	59.4
		ATGATCATGCAAGAGGAACTC	50.4	57.4	55.6	46.7	51.5
		AGACGGGATTTGTTAGGCACG	54.4	61.3	62.4	51.6	55.4
		CGTTAATGATGTATTAGGGGCATA	52.3	60.1	58.3	47.2	52.8
		TTTGTTAGGCACGATGATGGAT	51.1	58.4	60.5	49.1	53.2
		CACCTGACTGCTTTATTTCA	47.7	54.3	52.8	44.5	49.2
		TCTATCTCTGTAGCCCCTATTACG	55.7	63.5	57.2	49.4	55.9
		TCAGACGGGATTTGTTAGGC	51.8	58.4	58.9	48.7	52.2
		ATCTATCTCTGTAGCCCCTATTAC	54.0	61.8	53.4	47.3	53.3
		GGCAGATTCTAGACCTCCTGATGAAATAAA	58.9	68.0	65.6	53.3	59.7
		GCAAGAGGAACTCAGACGGG	55.9	62.5	61.1	51.4	55.3
		ACTCAGACGGGATTTGTTAGGC	54.8	62.1	60.7	51.3	55.0
		ACGGGATTTGTTAGGCACG	51.1	57.3	59.8	49.2	52.3
		TGTTTCCACCTCAATTAGTTTGAGAAGTGCCC	61.8	71.2	71.3	57.6	62.0
		TGATCATGCAAGAGGAACTCA	50.5	57.4	58.2	48.2	52.7
		AGTCAGGTGGTCTTATGCC	51.1	57.3	53.8	47.8	50.3
		AACTCAGACGGGATTTGTTAGG	53.0	60.3	58.5	49.0	53.2
		TGAAATAAAGCAGTCAGGTG	47.7	54.3	52.8	44.5	49.2
		TCTATCTCTGTAGCCCCTATT	50.5	57.4	51.5	45.8	51.0
	orfu	CGTCACACCAGTTACTTTTCG	52.4	59.4	57.2	48.0	52.8
		CCTAAACAAAATGAGCATGGC	50.5	57.4	58.5	46.9	51.5
		ATGCGCTATTTTCTACTGTTTTTG	50.6	58.4	58.0	47.3	53.8
		GGTGTTATTTGATGGCTGCATG	53.0	60.3	61.4	49.3	53.5
		AGCTCAATACGAAGTTTCACGC	53.0	60.3	59.8	50.2	55.7
		CAGAGCGCTGCATTTATCCTTATTG	56.0	64.1	64.1	51.3	59.1
		AGAGCGCTGCATTTATCCTTATTG	54.0	61.8	61.9	50.5	57.6
	zot	GCCCATAGACCACGATAA	48.0	53.8	53.1	44.6	47.9
		GCCACTTTAACCGCGCCAC	55.4	61.6	64.9	53.5	55.9
		CTGTCTTTCCACAGCGCCG	55.4	61.6	64.2	52.5	57.1
		TCGCTTAACGATGGCGCGTTTT	54.8	62.1	68.6	54.8	59.8
		CACTGTTGGTGATGAGCGTTATCG	57.4	65.2	64.9	52.7	58.3
		CAAAGCCGACCAATACAAAAACCAA	54.4	62.5	65.8	51.8	55.9
		GATATCTGCCTAACCACGCCTAAC	57.4	65.2	61.3	51.8	56.7
		AAGGTATCGAACACCACAAAGTGATTGAAATCCGGTAAC	64.5	74.7	74.7	59.6	64.5
	rstr	CTTCTCATCAGCAAAGCCTCCATC	57.4	65.2	64.6	52.5	58.6
		AGCCTCCATCAAAATGAATA	45.6	52.3	53.7	43.4	47.8
TCP Prophage	acfb	GATGAAAGAACAGGAGAGA	46.8	53.0	48.5	42.3	47.0
		TTTGTCTGAGCCGTATGTCG	51.8	58.4	58.7	48.8	53.7
		GAGCGTGCTTTATCATGGTCGAT	55.3	62.9	63.6	52.0	57.5
		CAGCAACCACAGCAAAACC	51.1	57.3	59.1	49.0	51.6
	acfc	CACTATTTGGGGCAAAAACG	49.7	56.4	59.3	46.6	50.6
	alda	GTCAATGGATGAAGCCACACAGTG	57.4	65.2	65.0	53.1	56.9
		AACCAAGGTGAGGTTTGTACC	52.4	59.4	56.9	49.2	50.8
	int	GATAAAGAGATCAAAGCC	43.5	49.3	45.6	38.3	43.1
		GAAGTAATGAAACCGATAAGTGG	51.7	59.3	55.9	46.0	51.2
		TGCTTTGTACCAGTCACAGATAG	53.5	61.1	56.1	49.0	54.1
		CCAATCCTTTGTGACGTTC	48.9	55.2	55.2	45.2	48.5

	**tagd**	ACCTACTTTAGGAAAAGAGCC	50.5	57.4	52.7	46.0	50.5
	**tcpd**	GCGCTTGGTAATCAAGAC	48.0	53.8	53.0	44.7	48.8
	**tcpe**	GCTCCTGACAATGGCTGTTTATTCA	56.0	64.1	64.6	52.6	57.4
	**tcpf**	ACTGTATAGCAAAGCATATTCAGAGA	53.2	61.6	57.0	49.0	56.0
		GAGTTCCACATGCAGAAACAGGA	55.3	62.9	62.6	52.0	55.7
		GACGCATACCCATCGACAGA	53.8	60.5	60.8	50.6	54.3
		GGAGTTATCTATGACCCTGTT	50.4	57.4	51.3	45.3	49.1
		AGGAGATGGAAGTGGTGTG	51.1	57.3	54.6	47.7	50.3
	**tcpi**	GGCAATAGTGTCGAGCTCGTTA	54.8	62.1	60.6	51.2	56.6
		TAGCCTTAGTTCTCAGCAGGCA	54.8	62.1	60.5	52.4	57.7
		CGACTGCTTTATCGCGAAGT	51.8	58.4	59.4	49.4	55.7
		GATGGTCAGATAAAAGAACGCAGG	55.7	63.5	62.2	50.5	56.1
	**tcpp**	TTAGCAAGGTTACCGGGGATAACAA	56.0	64.1	64.6	53.0	56.5
		TTGGATTGTTATCCCCGGTA	49.7	56.4	58.9	47.3	49.7
	**tcpq**	ACCGTGTAAATCAGCCCAAG	51.8	58.4	58.9	49.3	52.5
		AGCCAACTCAGTTAAAACTTGTTC	52.3	60.1	57.2	48.8	53.3
		GCACAAGGAGAGATGCACAA	51.8	58.4	58.8	49.5	53.3
	**toxt**	TACGCGTAATTGGCGTTGGGCAG	58.8	66.4	70.5	56.9	61.0
		ACAAATATCTGCCCAACG	45.8	51.6	53.2	43.8	47.4
		CTTGGTGCTACATTCATGG	48.9	55.2	53.7	44.7	48.9
		TCACCACAAATATCTGCCCA	49.7	56.4	58.7	48.2	50.8

Table S3: Proportion of valid primers according to a threshold set for the computed melting temperature. Three conditions were used to determine valid primers from the theoretical Tms: no threshold (Tm>0°C), Tm above 50°C (Tm>50°C) and Tm above 55°C (Tm>55°C). The published primers, which are not valid with no threshold of Tms (Tm>0), are primers that are not specific to *V. cholerae* and/or cannot detect every allele of their target gene. In some cases (e.g. *tcpA* or *ctxB*), none of many published primers is valid because the sequence of the target gene is very variable into the species and primers were not designed in conserved regions. Generally, this observation corresponds to very well studied genes, which were resequenced many times, increasing the number of different sequences in public databases.

**Gene**	**Published Primers**	**Valid Primers**		
		**Tm>0°C**	**Tm>50°C**	**Tm>55°C**
ace	8	4	4	4
acfb	6	6	5	4
acfc	1	1	1	1
alda	6	2	2	2
cep	3	2	2	2
ctxa	41	18	18	18
ctxb	23	0	0	0
int	5	4	4	4
orfu	9	7	7	7
rsta	8	0	0	0
rstb	3	0	0	0
rstr	3	2	2	2
taga	4	0	0	0
tagd	1	1	1	1
tcpa	27	0	0	0
tcpb	3	0	0	0
tcpc	1	0	0	0
tcpd	1	1	1	1
tcpe	1	1	1	1
tcpf	5	5	5	5
tcph	6	0	0	0
tcpi	7	4	4	4
tcpj	4	2	2	0
tcpp	12	2	2	2
tcpq	5	3	3	3
toxt	13	4	4	4
zot	24	8	8	8
**Total**	**230**	**77**	**76**	**73**

Table S4: Alignments of published primers and sequences of the *ctxB* gene.

Local alignments of 23 *ctxB* primers with their target sequences, perfect matches are shown as dots. Hyphens correspond to INDELs or missing part of the sequence (partial sequences). Columns: 1: strand, 2: Tm computed with OHM that is often slightly underrated, 3: alignment, 4: number of such target sequences. F is for forward primers, R for reverse ones.

	Tm	Alignment	Nbr.
F	49.8	ACTATCTTCAGCATATGCACATGG	22
	44.2	...................A....	1
	42.9	.................T......	1
F	47.4	TATCTTCAGCATATGCACAT	22
	39.9	.................A..	1
	38.2	...............T....	1
R	56.0	GAGGAGCTCCATGTGCATATGCTG	1
	44.2	....T.T.................	20
	38.5	....T.T.....T...........	1
	30.8	.T..T.T.......A.........	1
	0	....T.------------------	1
R	53.2	AGGTGTTCCATGTGCATATGC	21
	48.6	...........T.........	1
	44.8	T............A.......	1
	43.9	...A.C...............	1
F	51.4	TATGCACATGGAACACCTCAAA	20
	49.2	....................C.	1
	45.4	......A...............	1
	40.3	............G.T.......	1
	37.5	....T............A....	1
F	44.7	ACACCTCAAAATATTACTGA	19
	43.3	...........C........	1
	43.1	C	1
	38.9	........C..........C	1
	38.3	.....A..............	1
	34.2	G.T.................	1
F	50.1	ACTGATTTGTGTGCAGAATACCAC	15
	48.9	....C...................	2
	48.8	......................C.	3
	46.4	..................G.....	1
	44.7	...................CA...	1
	36.8	..............G......A..	1
	32.9	........A.....G.....T...	1
R	49.7	GGTATTCTGCACACAAATCAG	18
	48.5	...C.................	1
	44.3	.TG..................	1
	44.3	.................G...	2
	43.6	T......C.............	1
	34.3	.A.....C.....T.......	1

	Tm	Alignment	Nbr.
F	42.1	AGATATTTTCGTATACAGAATCTCTAG	20
	38.6	.......G...................	4
R	50.9	TGATAGCCATCTCTCTTTTTCCAG	22
	47.5	................G.......	2
R	48.4	GATAGCCATCTCTCTTTTTC	22
	43.4	...............G....	2
F	51.0	AAAGCGATTGAAAGGATGAAGG	22
	49.1	.....................A	1
	48.8	...........C..........	1
F	50.7	GCGATTGAAAGGATGAAGGATAC	22
	48.7	........C..............	1
	45.6	..................ACCGA	1
F	52.4	GCGATTGAAAGGATGAAGG	22
	50.0	..................A	1
	49.5	........C..........	1
F	52.2	TGAAAGGATGAAGGATACCC	22
	49.4	...C................	1
	32.7	.............ACCGAT.	1
R	54.8	TCCTCAGGGTATCCTTCATC	23
	36.1	.......ATCGGT.......	1
R	52.4	GATATGCAATCCTCAGGGTATCC	22
	47.4	....A..................	1
	38.4	................ATCGGT.	1
R	49.1	GCTTCAGTAAGATATGCAATCCTCAG	21
	46.4	......T...................	1
	44.9	..............A...........	1
	42.9	....TT....................	1
R	48.8	CGCATGAGGCGTTTTATTATTC	23
	37.8	.....T...T........G...	1
R	56.2	TAATTGCGGCAATCGCATGAGGCGT	23
	45.6	..................T...T..	1
R	59.1	ATTGCGGCAATCGCATGAGGCGT	23
	47.5	................T...T..	1
R	62.2	TGCGGCAATCGCATGAGGCG	23
	48.4	..............T...T.	1
R	52.7	GCCATACTAATTGCGGCAATCGCATG	23
	51.0	.........................T	1

Table S5: primers sets for *ctxA, ctxB* and *tcpA* genes.

Columns: 1: gene name, 2: forward primer, 3: reverse primer, 4: minimal melting temperature of the pair of primers (°C), 5: method used for the design. “Manual” corresponds to a design using the alignment of every genetic variant of the gene and a manual selection of conserved regions. Since Primaclade provided a large number of forward primers (and not 4 couples as Prifi), only partial Primaclade results are shown. The third column is filled by a double hyphen (--) for Primaclade, because this software returns only forward primers and not a couple of primers. For *ctxA,* only primers corresponding to valid published primers (and thus experimentally tested) were selected from the output of Primaclade.

Gene	Forward sequence	Reverse Sequence	Tm	Method
	TCGTATACAGAATCTCTAGCTGGAAA	GCCATACTAATTGCGGCAATCGC	55.0	Manual
	TCGTATACAGAATCTCTAGCTG	ATTTGCCATACTAATTGCGGC	59.4	Prifi
	GGTGTTTTTTTAYAGTTTTACTATCTTCAGC	ATTTGCCATACTAATTGCGGC	59.4	Prifi
	GGTGTTTTTTTAYAGTTTTACTATCTTCAGC	AATCGCTTTTTTTGTRAATCTATATGTTGAC	59.2	Prifi
	TCGTATACAGAATCTCTAGCTG	AATCGCTTTTTTTGTRAATCTATATGTTGAC	59.2	Prifi
	TACAGAATCTCTAGCTGGAAAMAGAG	--	59.7	Primaclade
ctxB	ATCTCTAGCTGGAAAMAGAGAGATG	--	59.6	Primaclade
	TAGCTGGAAAMAGAGAGATGGCTAT	--	61.0	Primaclade
	GGAAAMAGAGAGATGGCTATCATTAC	--	59.9	Primaclade
	TTAAGAATGGTGMAAYTTTTCAAGTA	--	61.4	Primaclade
	AATGGTGMAAYTTTTCAAGTAGAAG	--	60.4	Primaclade
	AAAAAGCGATTGAMAGGATGAAG	--	60.9	Primaclade
	AGCGATTGAMAGGATGAAGR	--	59.3	Primaclade
tcpA	CRCGATAAGAAAACMGGTCAAGAGG	CCGACRCCTTGTTGRTATTTTCTC	55.6	Manual
	CATTCTACTTATTATATATATGTTATAGCCACTGC	GGTATTCGTCAAGGAATTTTACACCTAG	59.5	Prifi
	AAATGAATATCAACCTTTATGATCATGCAAG	CAAGGAATTTTACACCTAGACTTTGGG	59.8	Prifi
	AAATGAATATCAACCTTTATGATCATGCAAG	ATGATCTTGGAGCATTCCCAC	59.8	Prifi
	TTTCCACCTCAATTAGTTTGAGAAGTG	ATGATCTTGGAGCATTCCCAC	59.8	Prifi
	ATGATCATGCAAGAGGAACTC	--	57.4	Primaclade
	CGTTAATGATGTATTAGGGGCATA	--	60.1	Primaclade
ctxA	CACCTGACTGCTTTATTTCA	--	54.3	Primaclade
	TCTATCTCTGTAGCCCCTATTACG	--	63.5	Primaclade
	TCAGACGGGATTTGTTAGGC	--	58.4	Primaclade
	ACGGGATTTGTTAGGCACG	--	57.3	Primaclade
	TGATCATGCAAGAGGAACTCA	--	57.4	Primaclade
	TGAAATAAAGCAGTCAGGTG	--	54.3	Primaclade
	TCTATCTCTGTAGCCCCTATT	--	57.4	Primaclade

Table S6: Examples of possible improvements for *ctxB* PCR primers.

Local alignments of primers with their target sequences, perfect matches are shown dots. Shorter primers contained into these primers are not shown. Columns: 1: Tm computed with OHM that is often slightly underrated, 2: consensus Tm computed with dnaMATE, 3: alignments, 4: number of target sequences. Primers can be improved by adding ambiguities shown in bold. Note that for the last primer, the last position could be simply removed.

Tm (°C)	Cons. Tm (°C)	Sequence alignment	Targets
	52.7	ACTATCTTCAGCATATGCACATGG	
49.8		....	21
44.2		...................A....	1
42.9		.................T......	1
44.9 – 49.8	50.1 – 53.7	ACTATCTTCAGCATATG**Y**A**M**ATGG	24
	50.3	AGATATTTTCGTATACAGAATCTCTAG	
42.2		....	20
38.6		.......G...................	4
42.2 – 43.9	50.3 – 51.3	AGATATT**K**TCGTATACAGAATCTCTAG	24
	52.4	TGATAGCCATCTCTCTTTTTCCAG	
50.9		....	22
47.5		................G.......	2
50.9 – 53.0	52.4 – 53.8	TGATAGCCATCTCTCT**K**TTTCCAG	24
	50.9	AAAGCGATTGAAAGGATGAAGG	
51.4		....	21
49.1		.....................A	1
48.8		...........C..........	1
50.0 – 53.1	50.9 – 53.1	AAAGCGATTGA**M**AGGATGAAG**R**	24
	61.2	TAATTGCGGCAATCGCATGAGGCGT	
56.2		....	23
45.6		..................T...T..	1
51.9 – 56.2	57.1 – 61.2	TAATTGCGGCAATCGCAT**K**AGG**Y**GT	24
	59.0	GCCATACTAATTGCGGCAATCGCATG	
52.7		....	23
51.1		.........................T	1
51.7 – 52.7	58.4 – 59.0	GCCATACTAATTGCGGCAATCGCAT**K**	24

Table S7: Examples of possible improvements for *tcpA*.

Local alignments of primers with their target sequences, perfect matches are shown by dots. Shorter primers contained into these primers are not shown. Columns: 1: Tm computed with OHM that is often slightly underrated, 2: consensus Tm computed with dnaMATE, 3: alignments, 4: number of target sequences. Primers can be improved by adding ambiguities shown in bold.

Tm (°C)	Cons. Tm (°C)	Sequence alignment	Targets
	51.5	ATGCAATTATTAAAACAGCTTTTTAAG	
39.5		....	43
37.9		.........C.................	3
39.5 – 41.1	51.5 – 52.4	ATGCAATTA**Y**TAAAACAGCTTTTTAAG	46
	55.1	CACGATAAGAAAACCGGTCAAGAGG	
52.5		....	44
47.9		.G.......................	1
45.3		..............A..........	1
50.5 – 52.5	55.1 – 57.5	C**R**CGATAAGAAAAC**M**GGTCAAGAGG	46

Table S8: Examples of possible improvements for other genes.

Local alignments of primers with their target sequences, perfect matches are shown dots. Columns: 1: gene name, 2: Tm computed with OHM that is often slightly underrated, 3: consensus Tm computed with dnaMATE, 4: alignments, 5: accession number of target sequences. Primers can be improved by adding ambiguities shown in bold.

Gene	Tm (°C)	Cons. Tm (°C)	Sequence alignment	Targets
cep		53.1	AACCCCGAGTGAAAGCGTG	
	58.1		....	CP001486
	54.4		...............G...	CP001236, AF220606
	58.1 – 58.3	51.7 – 53.1	AACCCCGAGTGAAAG**S**GTG	
zot		51.6	AACCCGTTTCACTTCTACCCA	
	54.1		....	CP001486, M83563
	47.7		.G.....C.............	AF220606
	54.1 – 59.5	51.6 – 55.5	A**R**CCCGT**Y**TCACTTCTACCCA	
tcpQ		56.6	GAGGACTGTTCTGCAATCTGCTCAT	
	54.1		....	X64098, FJ209009, X74730, FJ209005, FJ209007
	48.2		.............T...........	FJ209006, FJ209008, FJ209010
	51.5 – 54.1	53.9 – 56.6	GAGGACTGTTCTG**Y**AATCTGCTCAT	
orfU		58.7	AACTCGGTCTCGGCCTCTCGTATC	
	58.8		....	AE003852, AF220606
	54.6		.................G......	CP001235.PE1548
	58.8 – 60.5	58.7 – 61.2	AACTCGGTCTCGGCCTC**K**CGTATC	
tcpB		51.7	CGTTGGCGGTCAGTCTTG	
	56.5		....	FJ209005, FJ209007, CP001235, FJ209010, FJ209006, FJ209009, X64098, FJ209011, X74730
	53.4		T.................	FJ209008
	52.0		.......A..........	FJ209004
	55.2 – 57.9	46.5 – 51.5	**Y**GTTGGC**R**GTCAGTCTTG	

Table S9: Strains, biovars and serotypes of nucleic sequences.

Columns: 1: gene name, 2: accession number , 3: number of other sequences identical to or contained in the sequence, 4: species names, 5: biovar, serotype and strain information.

	Accession number	Duplicates	Species	Biovar, Serotype and Strain
ctxA	D30052.CTXA	30	Vibrio cholerae	O1 0402, O1 0403, O1 0404, O1 0405, O1 0406, O1 0407, O1 0408, O1 0409, O1 0410, O1 0502, O1 0503, O1 0504, O1 0505, O1 0506, O1 0507, O1 0508, O1 0601, O1 0602, O1 0603, O1 0604, O1 0605, O1 0606, O1 0702, O1 0706, O1 0709, O105 571-88, O139 0401, O141 203-93, O37 1322-69, O37 S7
	FJ748608.CTXA	0	Vibrio cholerae	non O1/O139 J31W
	AJ575590.TOXA	0	Vibrio cholerae	Inaba
	X00171.PE1	1	Vibrio cholerae	2125
	X58786.PE1	14	Vibrio cholerae	Classical 569B, El Tor 2125, El Tor O1 B33, El Tor O1 N16961, El Tor O1 VC44, Inaba O1 VC04, MJ-1236, MTCC 3906, O1 O395, O139 JS9803, O139-Bengal 1854
	AF175708.CTXA	0	Vibrio cholerae	KNIH002
	AF390572.CTXA	10	Vibrio cholerae	Classical O1 GP8, Classical O1 H218, Classical O1 NIH35A3, Classical O1 NIH41, Classical O1 V154, Classical O1 VC44, El Tor O1 B33, El Tor O1 CO457, El Tor O1 VC106, El Tor O1 VC20, O27 365-96
	AY376268	0	Vibrio cholerae	non O1/O139 F
	AY376267	0	Vibrio cholerae	non O1/O139 B
	DQ132785	0	Vibrio cholerae	
hapA	CP001486.PE458	3	Vibrio cholerae	El Tor O1 N16961, MJ-1236, O1 M66-2, Ogawa O1
	M59466.PE1	0	Vibrio cholerae	DH5-alpha
	CP001236.HAP	1	Vibrio cholerae	Ogawa O1 O395
	AB435238	0	Vibrio mimicus	ES-39
	AB071709.VFPA	0	Vibrio fluvialis	AQ0005
	M64809.PE1	0	Vibrio proteolyticus	
	EU675309	0	Vibrio tubiashii	RE22
	FJ455120	0	Vibrio tubiashii	00-90-6
	FJ455119	0	Vibrio tubiashii	X00-12-1; RE98
	FJ455121	0	Vibrio tubiashii	ATCC 19105
	U50548.PE1	0	Vibrio vulnificus	
hlyA	CP000626.HLYA	0	Vibrio cholerae	Ogawa O1 O395
	AJ576090.HLYA	0	Vibrio cholerae	Inaba
	Y00557.HLYA	2	Vibrio cholerae	017, El Tor O1 N86, O1 M66-2
	CP001486.PE34	7	Vibrio cholerae	ATCC 14035, CIP104154, Clin2200, El Tor N16961, Env25, M793, MJ-1236, O139
	M36855.PE1	0	Vibrio cholerae	
	GU809235	0	Vibrio cholerae	Env40
	GU586279	0	Vibrio cholerae	Env8
	GU809234	0	Vibrio cholerae	Env18Q
	GU586281	0	Vibrio cholerae	Env34
	AF194418.HLYA	0	Vibrio cholerae	3509
	GU593975	0	Vibrio cholerae	Env4Q
	D58374.PE1	1	Vibrio cholerae	N037
	AY427780.PE1	0	Vibrio cholerae	El Tor non O1/O139
	GU586277	0	Vibrio cholerae	Env31Q
	GU230682	0	Vibrio harveyi	STD 3-0953
	GU230681	0	Vibrio harveyi	STD 3-0949
	GU137288.VMHA	0	Vibrio mimicus	ATCC 33653
	U68271.VMHA	0	Vibrio mimicus	ATCC 33653
	GU137289.VMHA	0	Vibrio mimicus	03-4472g2
	GU137291.VMHA	0	Vibrio mimicus	05-3478
	GU137290.VMHA	0	Vibrio mimicus	CDC08-2487
	EF187438	0	Vibrio mimicus	HX4
	FJ222406	0	Vibrio mimicus	
	AF348455	0	Vibrio mimicus	
ompU	CP001485.PE2734	2	Vibrio cholerae	El Tor O1 N16961, MJ-1236, O1 M66-2
	CP001235.OMPU	2	Vibrio cholerae	Ogawa O1 O395
	AF253529	0	Vibrio cholerae	El Tor
	DQ356330	0	Vibrio mimicus	04-14
	DQ356331	0	Vibrio mimicus	HX4
	DQ846741	0	Vibrio mimicus	04-5

	EU285491	0	Vibrio tubiashii	ATCC 19109
	DQ846743	0	Vibrio mimicus	04-13
	EU285490	0	Vibrio tubiashii	ATCC 19105
	AE016795.PE1557	0	Vibrio vulnificus	CMCP6
	BA000037.VV2720	0	Vibrio vulnificus	YJ016
	DQ980545	0	Vibrio vulnificus	VV 7-00
	DQ980544	0	Vibrio vulnificus	NCIMB 2136
	CP001805.PE989	0	Vibrio sp.	EX25
	CP000789.PE3292	0	Vibrio harveyi	ATCC BAA-1116; BB120
	FJ919231	0	Vibrio harveyi	zj2008
	CP001806.PE1228	0	Vibrio sp.	EX25
	CP000790.PE374	0	Vibrio harveyi	ATCC BAA-1116; BB120
	AB441168	0	Vibrio cholerae	O21
	FJ462453	0	Vibrio cholerae	non O1/O139 08-5742
	FJ462448	0	Vibrio cholerae	non O1/O139 08-5738
	FJ462449	0	Vibrio cholerae	non O1/O139 08-5737
	FJ462446	0	Vibrio cholerae	non O1/O139 08-5735
	FJ462447	0	Vibrio cholerae	non O1/O139 08-5739
	CP001486.PE457	3	Vibrio cholerae	ATCC 55056, El Tor O1 N16961, MJ-1236, O1 M66-2
	X51948.PE1	0	Vibrio cholerae	
	AF001009.OMPW	0	Vibrio cholerae	
	CP001236.OMPW	1	Vibrio cholerae	Ogawa O1 O395
ompW	FJ462450	0	Vibrio cholerae	ATCC 27070
	BA000032.VPA0096	1	Vibrio parahaemolyticus	O3:K6, ATCC 17802
	DQ425109	0	Vibrio parahaemolyticus	ZJ2003
	AY944132	0	Vibrio alginolyticus	
	DQ075316	0	Vibrio alginolyticus	
	GQ891116	0	Vibrio alginolyticus	HY9901
	CP001806.PE902	0	Vibrio sp.	EX25
	CP000790.PE2148	0	Vibrio harveyi	ATCC BAA-1116; BB120
	FJ998268	0	Vibrio harveyi	ZJ2008
	FM954973.PE1234	0	Vibrio splendidus	LGP32
	M85198.STN	0	Vibrio cholerae	NRT-36
sto	M97591.STO	2	Vibrio cholerae	El Tor GP156, HN0375
	AF302048.PE1	0	Vibrio mimicus	
	AF030546	0	Vibrio cholerae	non O1/O139 #151
	DQ132784.PE1	1	Vibrio cholerae	non O1/O139 #208
	FJ209006.TCPA	0	Vibrio cholerae	O27 M1121
	AF414371	0	Vibrio cholerae	O36 VCE 22
	AF512421.TCPA	0	Vibrio cholerae	EVC O1 ZJ6061
	EU622531.TCPA	4	Vibrio cholerae	EVC O1 LN93094, EVC O1 LN93097, EVC O1 ZJ4042, HB84419, O1 ZJ59
	AF512423.TCPA	0	Vibrio cholerae	EVC O1 SD7743
	FJ209004.TCPA	1	Vibrio cholerae	O80 M1098, O77 8-76
	AF452571	0	Vibrio cholerae	O26 63
	FJ209009.TCPA	2	Vibrio cholerae	O37 1322-69, O37 V52, non O1/O139 M1618
	X64098.TCPA	0	Vibrio cholerae	Classical O1 Z17561
	M33514.TCPA	0	Vibrio cholerae	Classical Z17561
	AF315787	0	Vibrio mimicus	343CAS
	AB012946	0	Vibrio cholerae	
	AY056618.TCPA	0	Vibrio cholerae	GX95065
tcpA	EU622528.TCPA	0	Vibrio cholerae	O1 ZJ24
	AF452580	0	Vibrio cholerae	O191 366-96
	AF452573	0	Vibrio cholerae	O48 AQ1875
	EU622527.TCPA	0	Vibrio cholerae	O1 ZJ22
	EU362122	0	Vibrio cholerae	O56 A199
	GU797082	0	Vibrio cholerae	O56 A217
	EU622526.TCPA	0	Vibrio cholerae	O1 ZJ20
	FJ209005.TCPA	2	Vibrio cholerae	O105 577-88, O105 M1118, O4 VCE232
	AF208385	0	Vibrio cholerae	O44 SCE188
	AF390571	0	Vibrio cholerae	O27 365-96
	AF452574	0	Vibrio cholerae	O49 507-94
	EU622525.TCPA	0	Vibrio cholerae	ZJ109
	AF139626	0	Vibrio cholerae	O59 10259
	FJ209008.TCPA	1	Vibrio cholerae	O141 203-93, O141 M1593
	GU797083	0	Vibrio cholerae	O56 K216
	GU797084	0	Vibrio cholerae	non O1/O139 A213
	FJ209007.TCPA	1	Vibrio cholerae	O115 523-80, O115 M1567
	AY056619.TCPA	0	Vibrio cholerae	XJ90006

	DQ356010	0	Vibrio mimicus	HX4
	EU622529.TCPA	5	Vibrio cholerae	El Tor O139 JS9803, O1 ZJ25, O139 HLJ9803, O139 JS9801, O139 JX98108, O139 LN1997
	AF512411.TCPA	1	Vibrio cholerae	O139 GD9512, O139 NM1996
	EU649677.TCPA	0	Vibrio cholerae	Y1
	EU622532.TCPA	0	Vibrio cholerae	O1 ZJ65
	AF512408.TCPA	0	Vibrio cholerae	O139 XJ93131
	X74730.TCPA	29	Vibrio cholerae	EVC O1 FJ62168, EVC O1 GD1961, EVC O1 GD98224, EVC O1 GX9525, EVC O1 LN9337, EVC O1 SD76137,EVC O1 SD7783, EVC O1 SD7763, EVC O1 WUJIANG2, EVC O1 XJ73329, El Tor E7946, El Tor O1 C6706, El Tor O1 H1, El Tor O1 N16961, El Tor O1 SC8511, El Tor O1 SC98107, El Tor O1 SM115, El Tor O139 FJ98352, El Tor O139 JX94484, MJ-1236, O1 M2140, O1 M66-2, O1 ZJ47, O139 63-93(MO45), O139 FJ9510, O139 MO10, O139 SD01001, O37 CO130, O44 506-94, O53 8585
	AY461808	0	Vibrio cholerae	O139 XJ93006
	FJ209003.TCPA	0	Vibrio cholerae	O1 M794
	AY052831.PE1	0	Vibrio cholerae	SD95001
	EU622533.TCPA	1	Vibrio cholerae	O1 ZJ75, O8 V54
	AF452570	0	Vibrio cholerae	O8 153-94
	FJ209010.TCPA	0	Vibrio cholerae	non O1/O139 M1619
tcpI	L25659.TCPJ	3	Vibrio cholerae	Ogawa O1 O395
	X64098.TCPI	0	Vibrio cholerae	Classical O1 Z17561
	CP001485.PE2461	4	Vibrio cholerae	El Tor O1 H1, El Tor O1 N16961, MJ-1236, O1 M66-2
toxR	M21249.PE1	2	Vibrio cholerae	E7946
	FM202719	0	Vibrio cholerae	CECT 514T
	CP001235.TOXR	1	Vibrio cholerae	Ogawa O1 O395
	CP001485.PE2314	2	Vibrio cholerae	MJ-1236, O1 M66-2, El Tor O1 N16961
	GU230678.TOXR	0	Vibrio harveyi	STD 3-0953
	GU230677.TOXR	0	Vibrio harveyi	STD 3-0949
	EF693743.TOXR	0	Vibrio mimicus	1.1969
	AF170881	0	Vibrio mimicus	ATCC 33655
zot	M83563.ZOT	14	Vibrio cholerae	Classical O1 GP8, Classical O1 H218, Classical O1 NIH35A3, Classical O1 NIH41, Classical O1 V154, Classical O1 VC44, El Tor O1 B33, El Tor O1 CO457, El Tor O1 VC106, El Tor O1 VC20, Ogawa O1 O395
	CP001486.PE741	3	Vibrio cholerae	El Tor O1 N16961, KNIH002, MJ-1236, Ogawa O139
	AF207857	0	Vibrio mimicus	PT5
	AF220606.ZOT	0	Vibrio cholerae	El Tor O1 86015

Table S10: List of softwares available for the design of primers. "Speciality" describes if softwares can design primers for specific molecular methods and "Input" reports the several types of format compatible with each software. "Parameter settings" presents common parameters of softwares and the methods used to compute melting temperature (Tm). "Option" shows if a program supports degenerated positions (IUPAC code), checks specificity (BLAST of primers) and coverage (BLAST of input sequences) of primers, and allows using target sequences and non-target sequences. qPCR: quantitative PCR, RFLP: Randow Fragment Length PCR, SNP: Single Nucleotide Polymorphism.

		dnaMATE	dPrimer	GeneFisher-P	GeneFisher2	iCODEHOP	MuPlex	OHM	OligoCalc	PCRTiler	PriFi	Prim-SNPing	Primer-BLAST	Primer3	PrimerHunter	Primers4clades	Primique	PRISE	Pythia	QuantPrime	UniPrime2
Type	*De novo* primer design			•	•	•	•			•	•	•	•	•	•	•	•	•	•	•	•
	Primer properties determination	•	•			•		•	•												
Speciality	Long-Range PCRs																		•		
	Multiplex PCRs						•				•										
	qPCRs																			•	
	Regular PCRs		•	•	•	•				•		•	•	•	•	•	•	•	•		•
	RFLP											•									
	SNP Genotyping / Mutagenesis						•					•									
Input	GenBank ID					•							•								
	Multiple amino acid sequences			•	•	•															
	Multiple nucleic acid sequences	•	•	•	•		•	•		•	•	•			•	•	•	•			
	Reference SNP ID											•									
	Single amino acid sequence			•	•	•															
	Single nucleic acid sequence	•	•	•	•		•	•	•	•	•	•	•	•	•	•	•	•	•		•
Parameter settings	%GC			•	•		•		•			•		•	•			•	•		
	Amplicon size			•	•		•			•	•	•	•	•	•	•		•	•		•
	Primer size			•	•		•			•	•	•	•	•	•			•	•		
	Secondary structure			•	•	•	•		•		•	•		•	•			•	•		
	Substrate concentrations					•	•		•		•	•		•	•				•		
	Basic method (Tm)	•						•	•												
	Salt-adjusted method (Tm)	•				•		•	•												
	Nearest neighbor method (Tm)	•	•	•	•		•	•	•	•	•	•	•	•	•	•	•	•	•	•	•
Options	BLAST for input sequences			•		•	•														
	BLAST for primers									•		•	•								
	IUPAC code		•	•	•	•	•			•	•		•		•						
	Non-target sequences							•							•		•	•			
	Region selection					•					•			•					•		
